# Supplementary figures and images for: Genome instability footprint under rapamycin and hydroxyurea treatments
Source: PLoS Genet. 2023 Nov 6;19(11):e1011012. doi: 10.1371/journal.pgen.1011012 (PMC10653606; doi:10.1371/journal.pgen.1011012)

S1 Fig

A

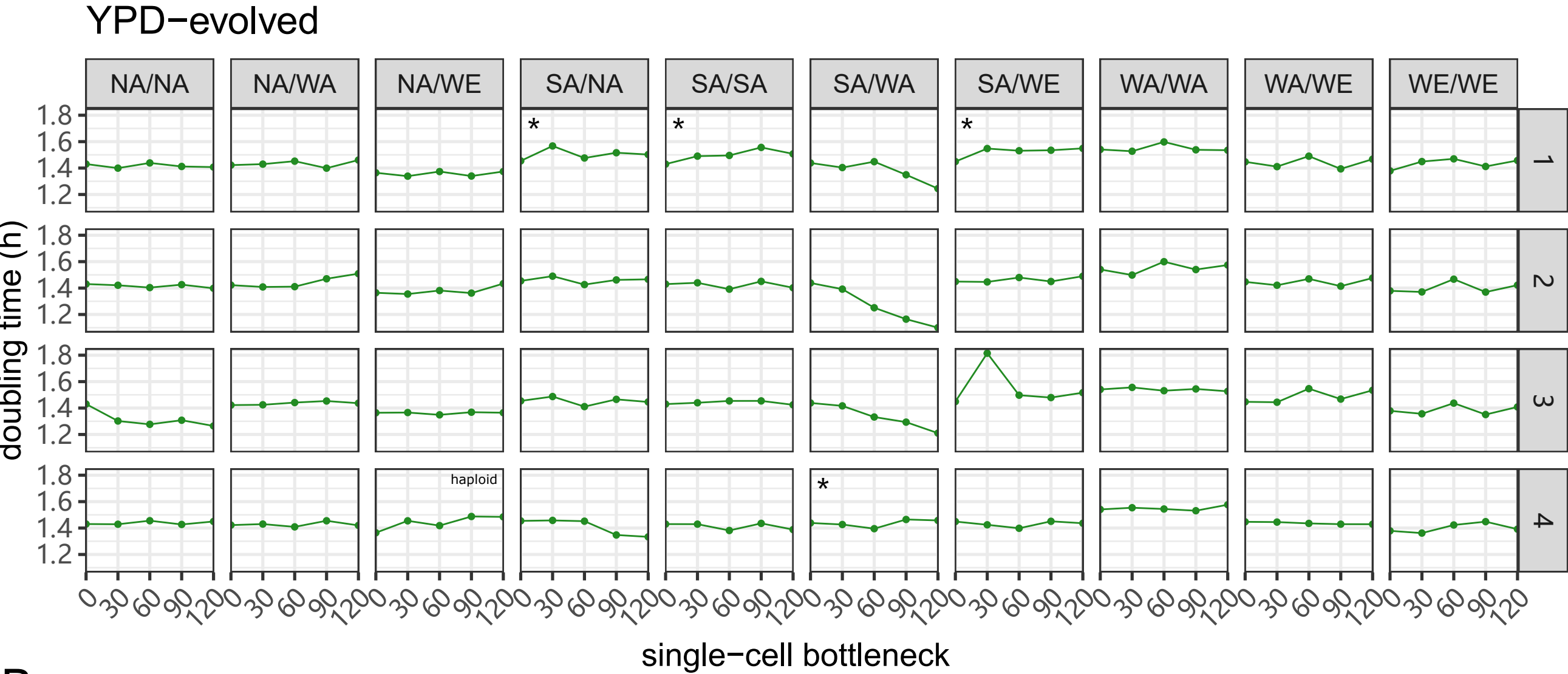

B

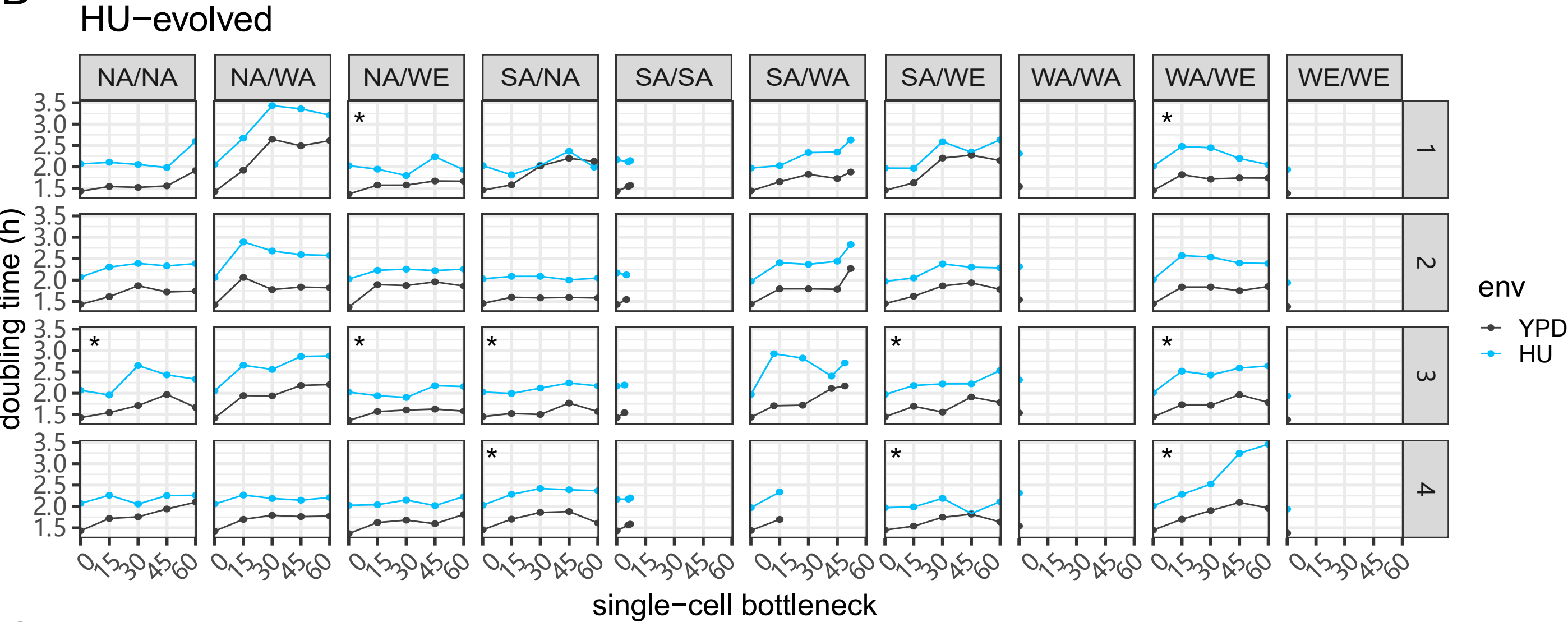

C

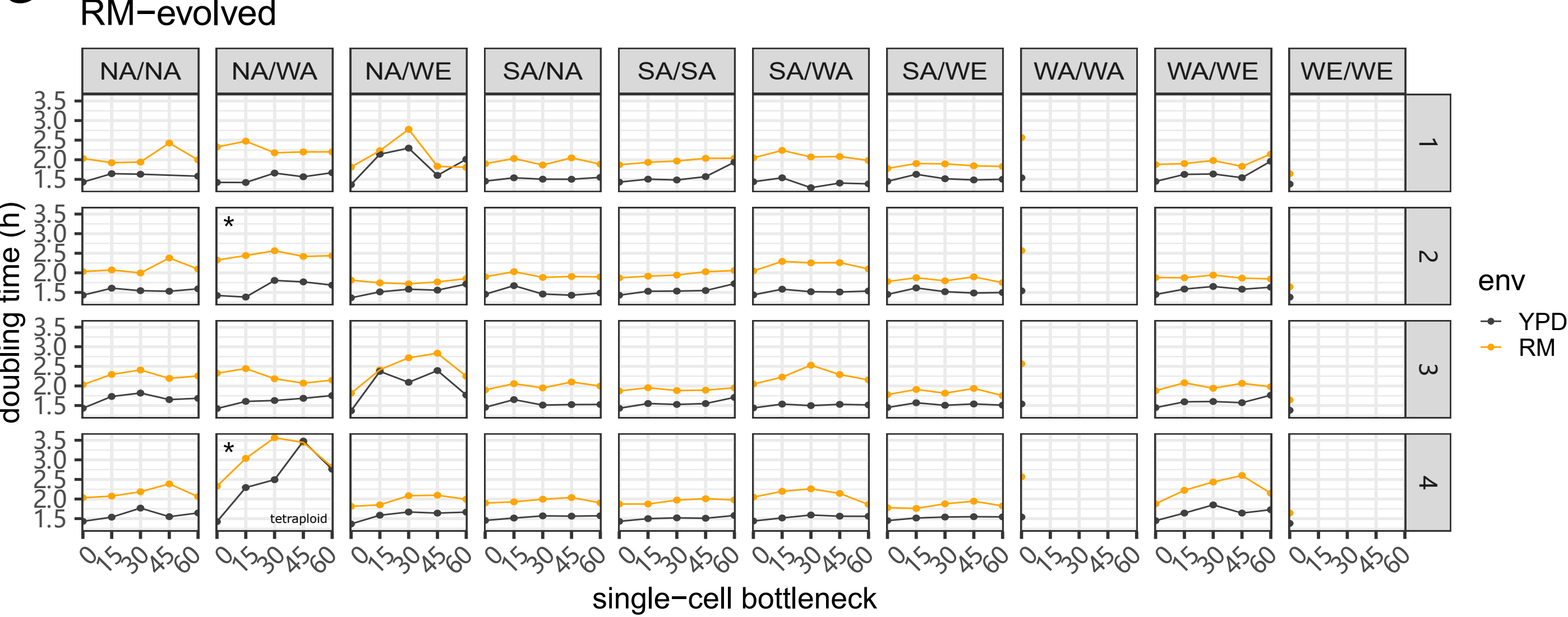

Supplement: S1 Fig — The doubling time dynamics of the (A) YPD-evolved, (B) HU-evolved, and (C) RM-evolved MALs across five time points during mutation accumulation. The green, blue, and orange dots show the doubling time (hours) measured in YPD, HU and RM condition respectively while the grey dots show the doubling time of drug-evolved MALs phenotyped in drug-free condition. The NA/WA-RM-4 line revealed strong growth defects in both RM and YPD conditions, consistent with its extensive unbalanced chromosome number (mean doubling time 2.82 h vs. 2.26 h of other NA/WA MALs in RM condition, 2.76 h vs. 1.70 h in YPD condition). “*” indicates mtDNA loss. (PDF) [file pgen.1011012.s009.pdf]

S2 Fig

A

YPD-evolved

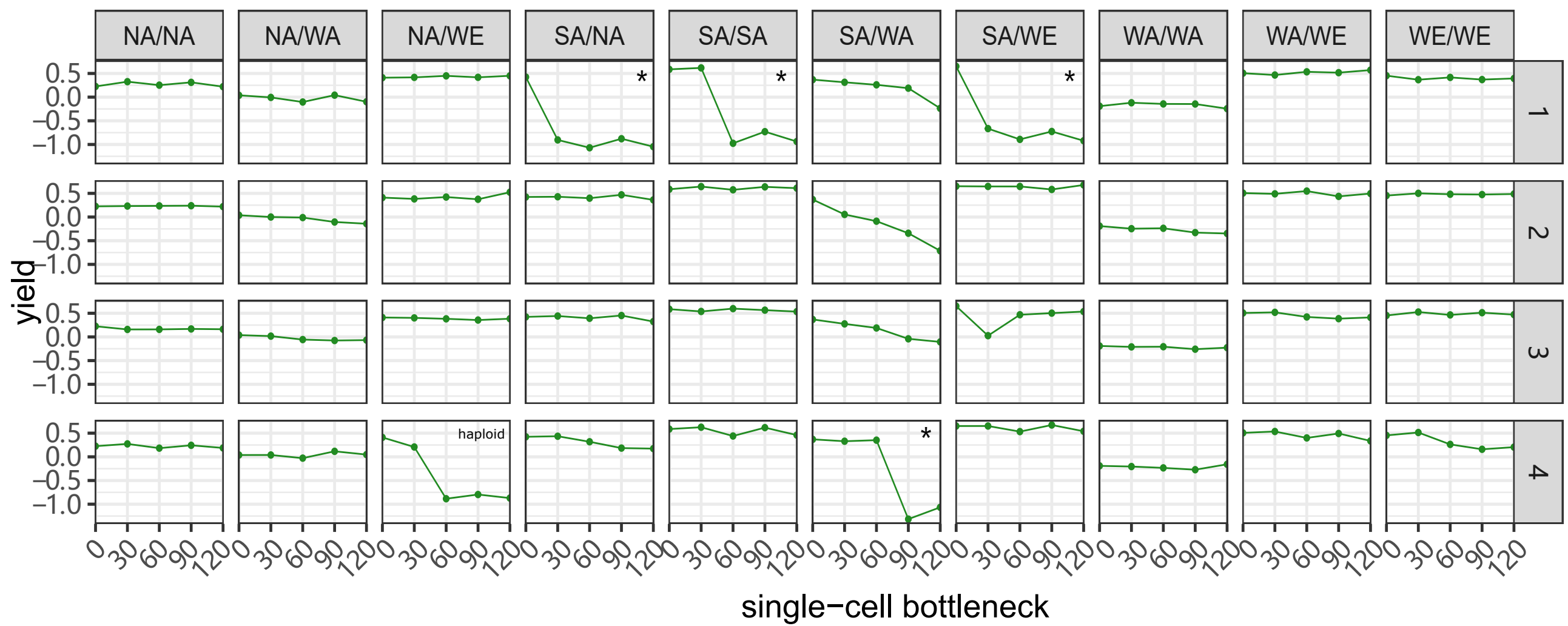

B

HU-evolved

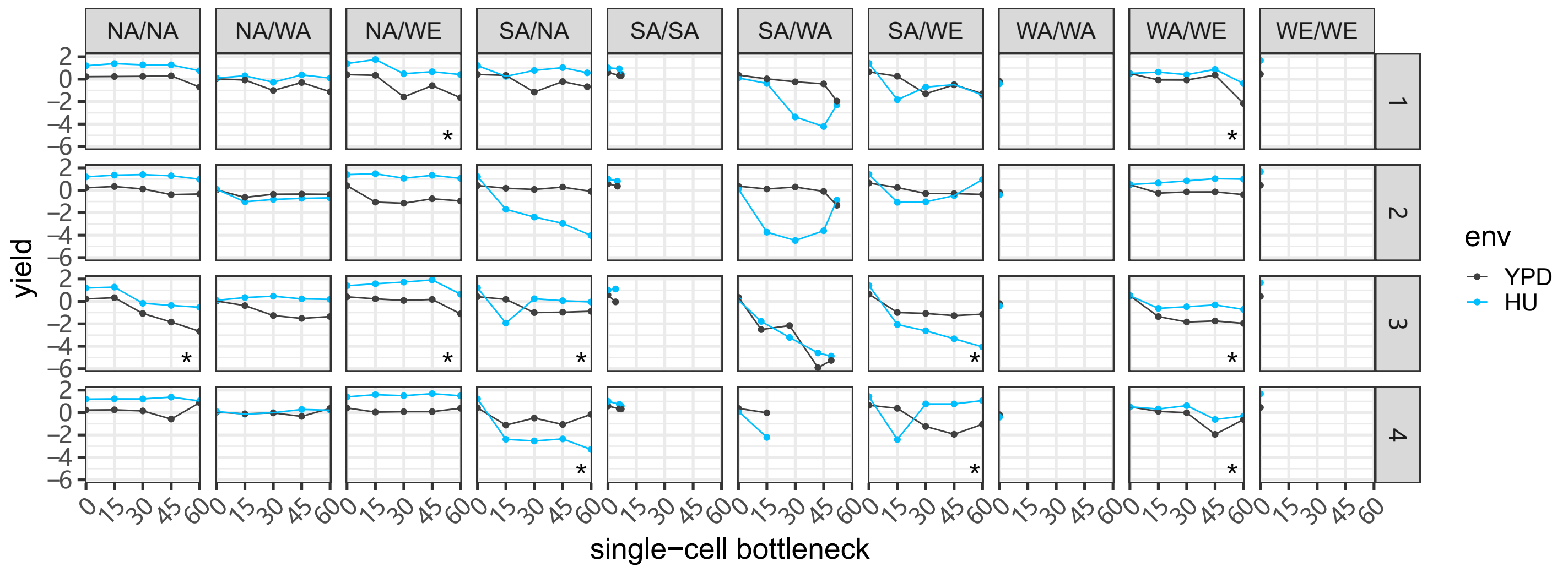

C

RM-evolved

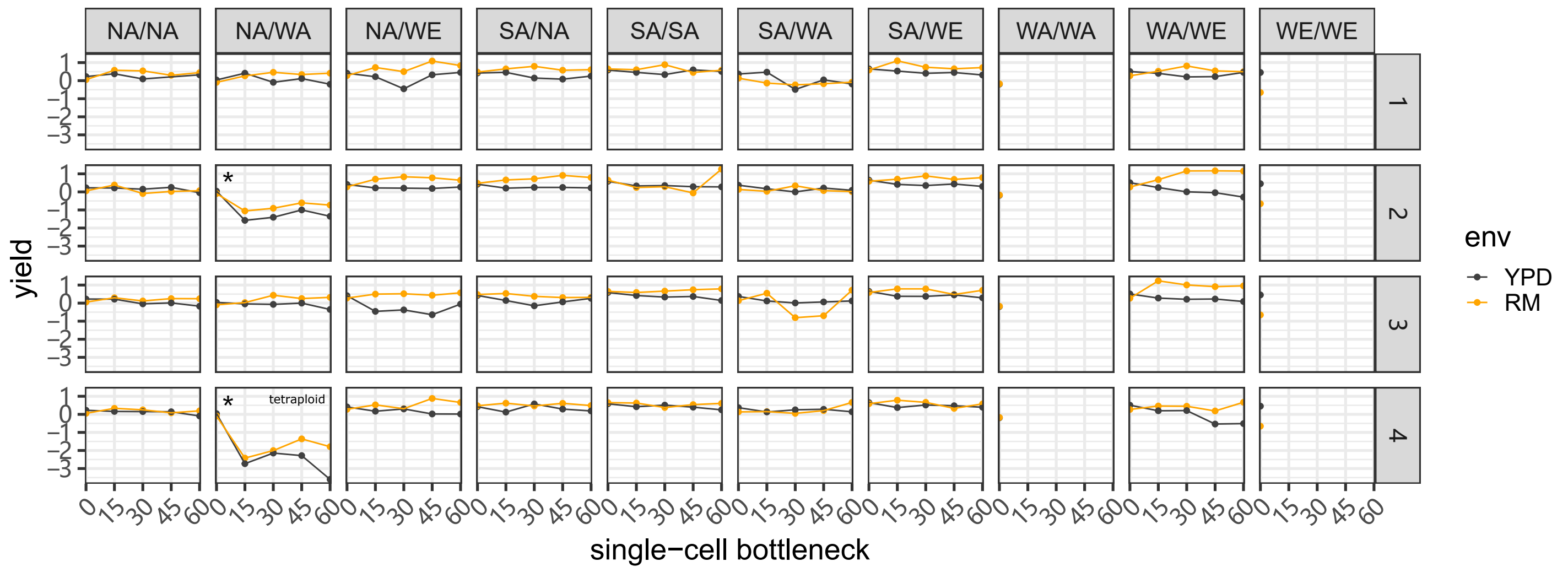

Supplement: S2 Fig — The dynamics of relative yield of all the (A) YPD-evolved, (B) HU-evolved, and (C) RM-evolved MALs across five time points during mutation accumulation. The green, blue, and orange dots show the relative yield measured in YPD, HU and RM condition respectively while the grey dots show the yield of drug-evolved MALs phenotyped in drug-free condition. “*” indicates mtDNA loss. (PDF) [file pgen.1011012.s010.pdf]

S3 Fig

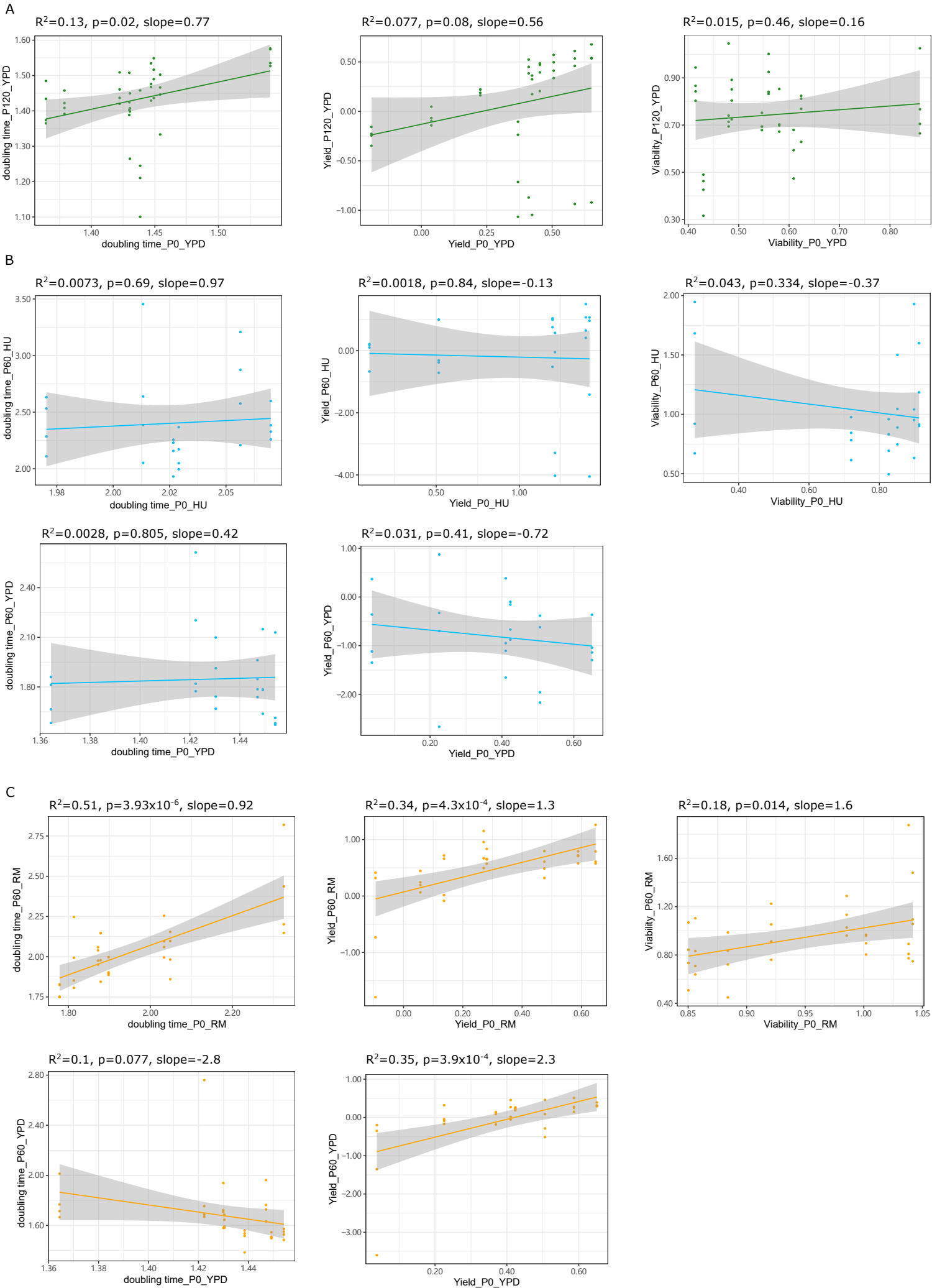

Supplement: S3 Fig — Phenotypic correlations between different time points and different conditions for MALs evolved in (A) YPD, (B) HU and (C) RM. For (A-C), left panel: doubling time correlation; middle panel: yield correlation; right panel: viability correlation. For (B) and (C), from up to bottom the correlation analysis includes comparison between the initial time point and end time point phenotyped in drugs (upper panel) and without drugs (bottom panel). (PDF) [file pgen.1011012.s011.pdf]

S4 Fig

A

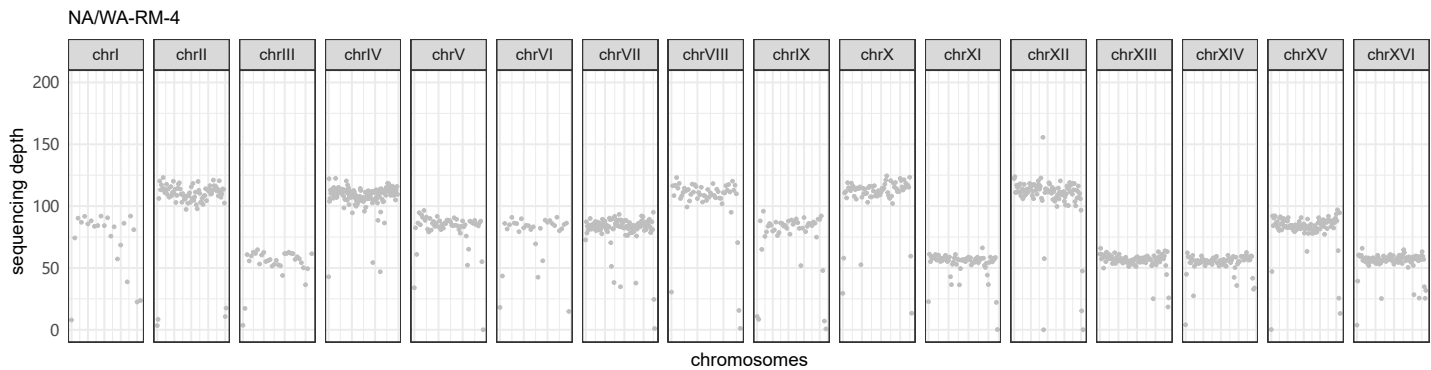

B

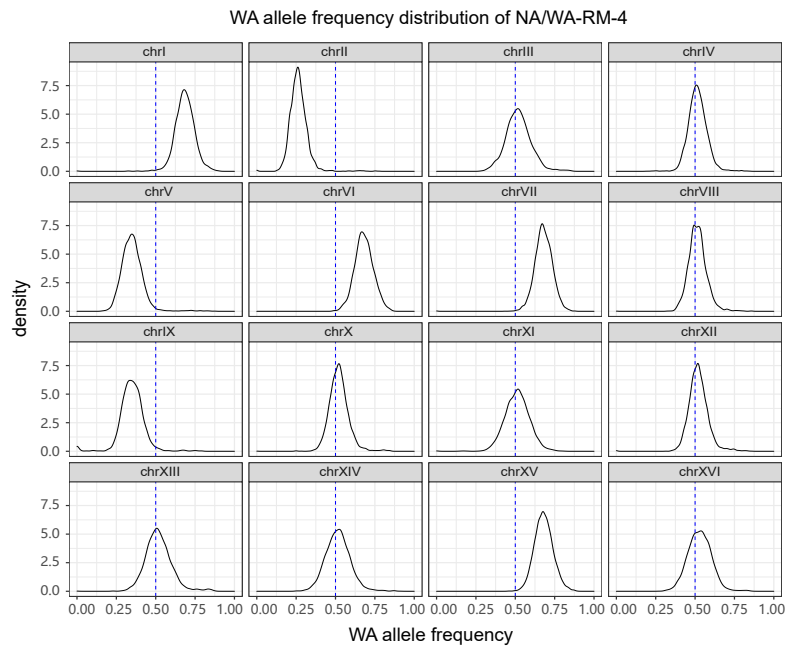

C

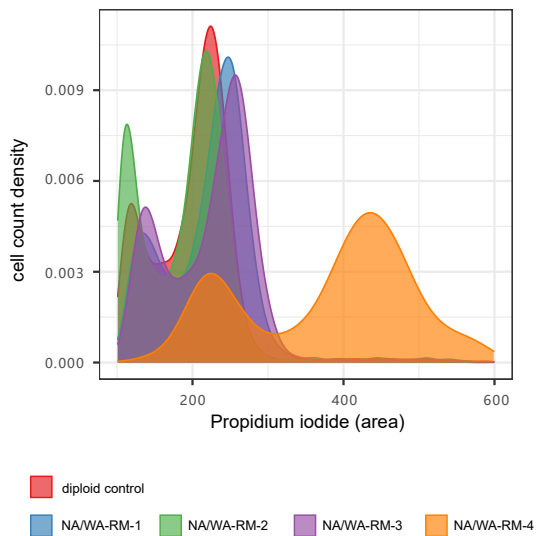

Supplement: S4 Fig — (A) Sequencing depth of NA/WA-RM-4 across all the chromosomes. (B) WA allele frequency distribution of NA/WA-RM-4 on each chromosome. Chromosomes II and XV are present in four copies but with an unbalanced WA allele frequencies (1:3 and 3:1 respectively). Such scenario is consistent with loss of one copy and subsequent resynthesis using the other parent homolog as template. We described a similar situation in a MAL initiated with a natural S. cerevisiae x S. paradoxus hybrid that also experienced WGD and subsequent chromosome loss and re-synthesis cycles, suggesting that this mechanism is prevalent in tetraploids (D’Angiolo 2020). (C) Ploidy flow cytometry profiles for all the four NA/WA-RM- evolved lines. (PDF) [file pgen.1011012.s012.pdf]

# S5 Fig

A

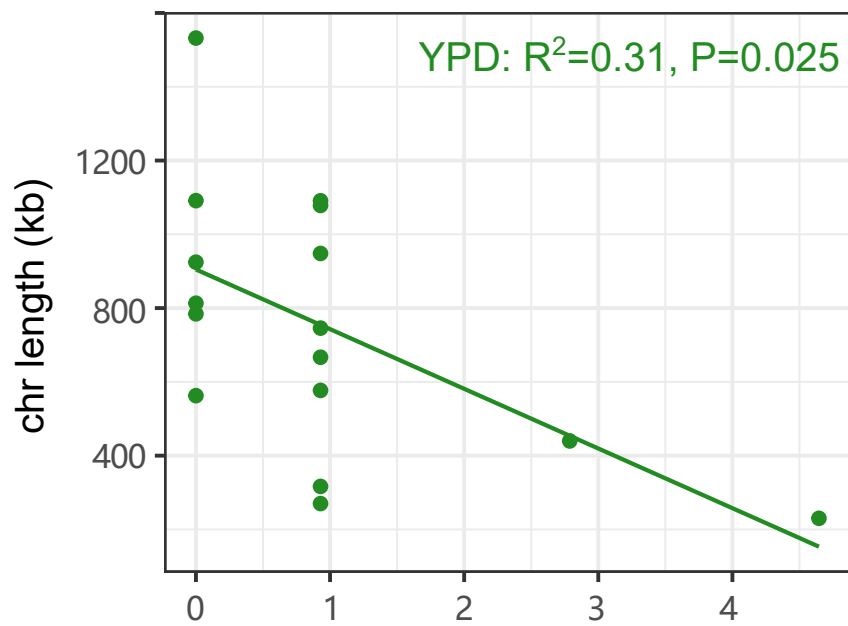

B

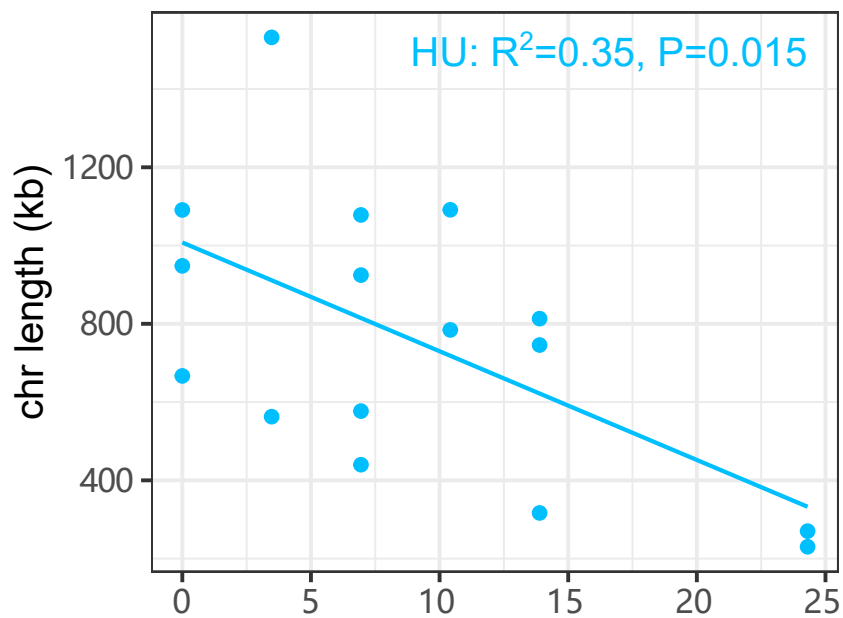

C

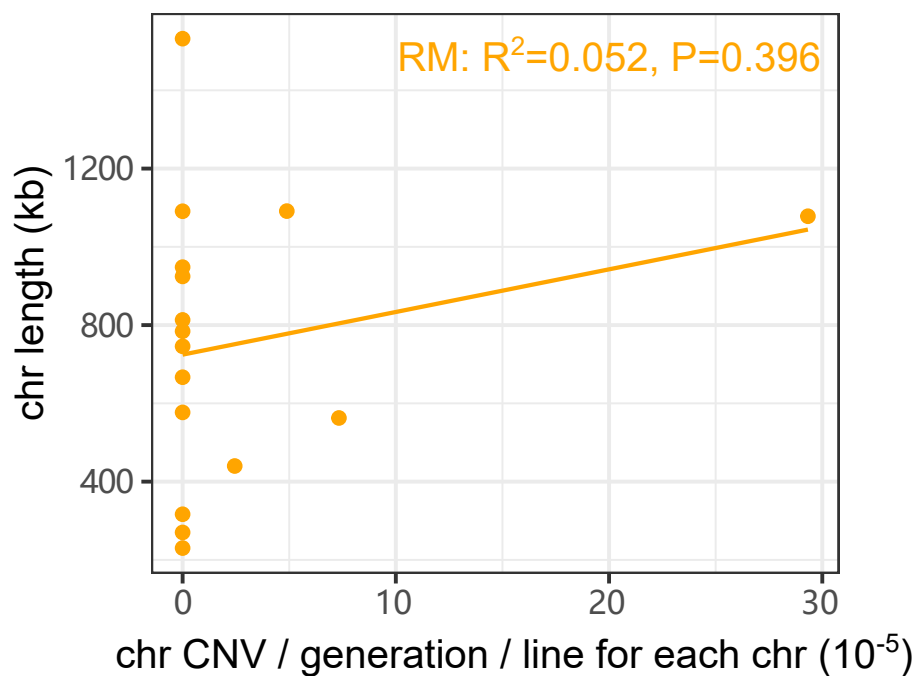

Supplement: S5 Fig — The correlation of chromosome length and chromosomal CNV across MALs in (A) YPD, (B) HU and (C) RM condition. (PDF) [file pgen.1011012.s013.pdf]

# S6 Fig

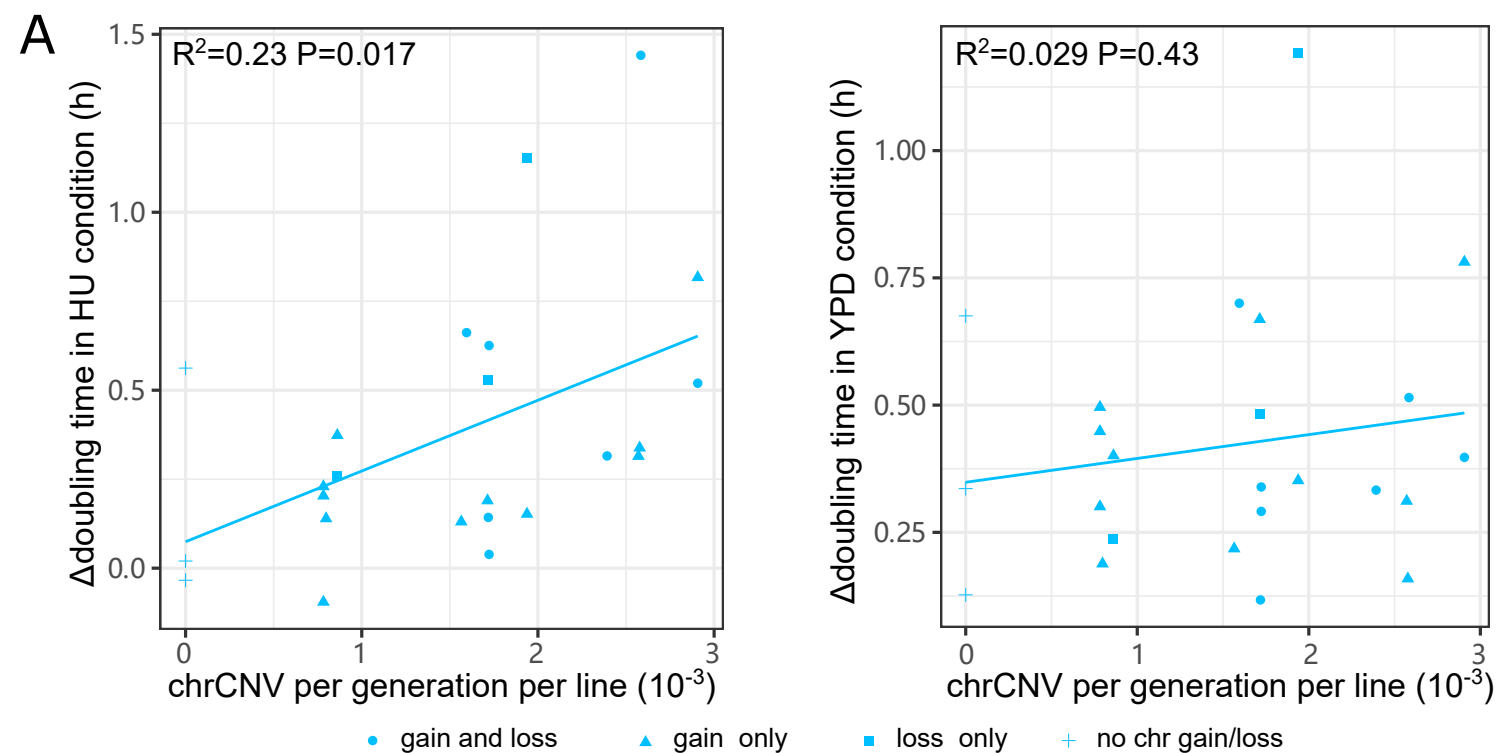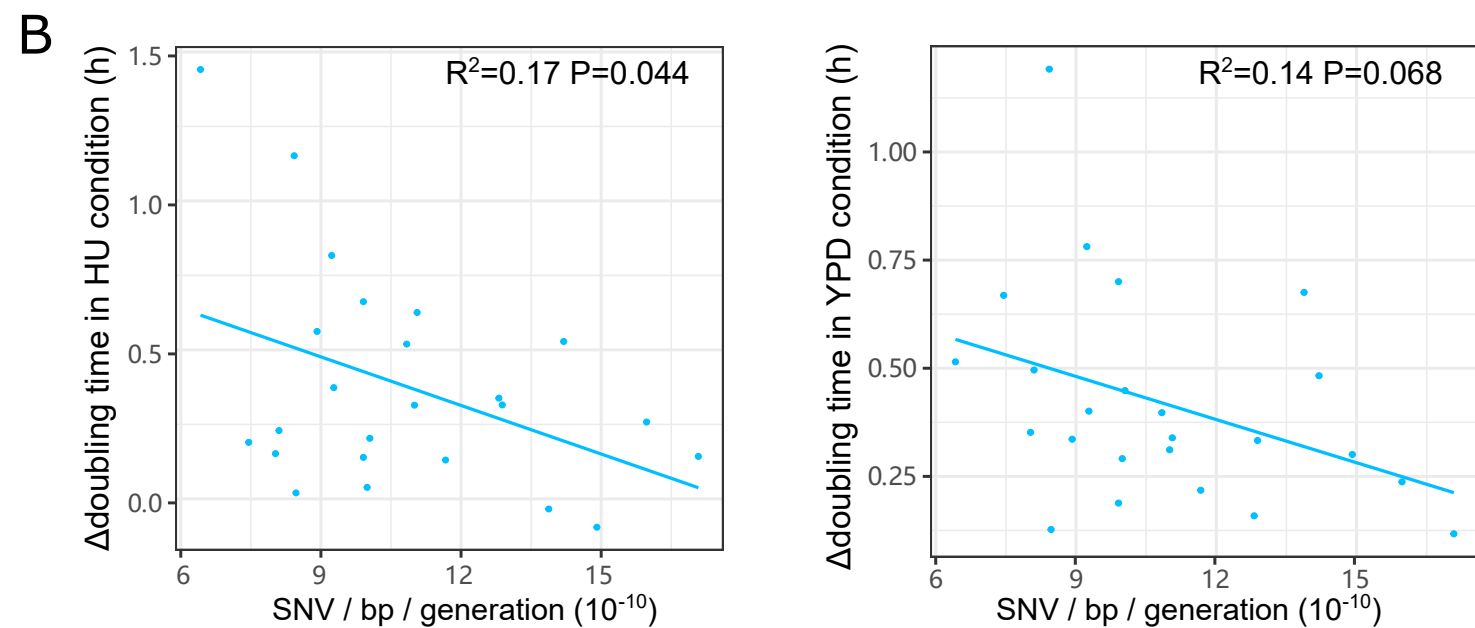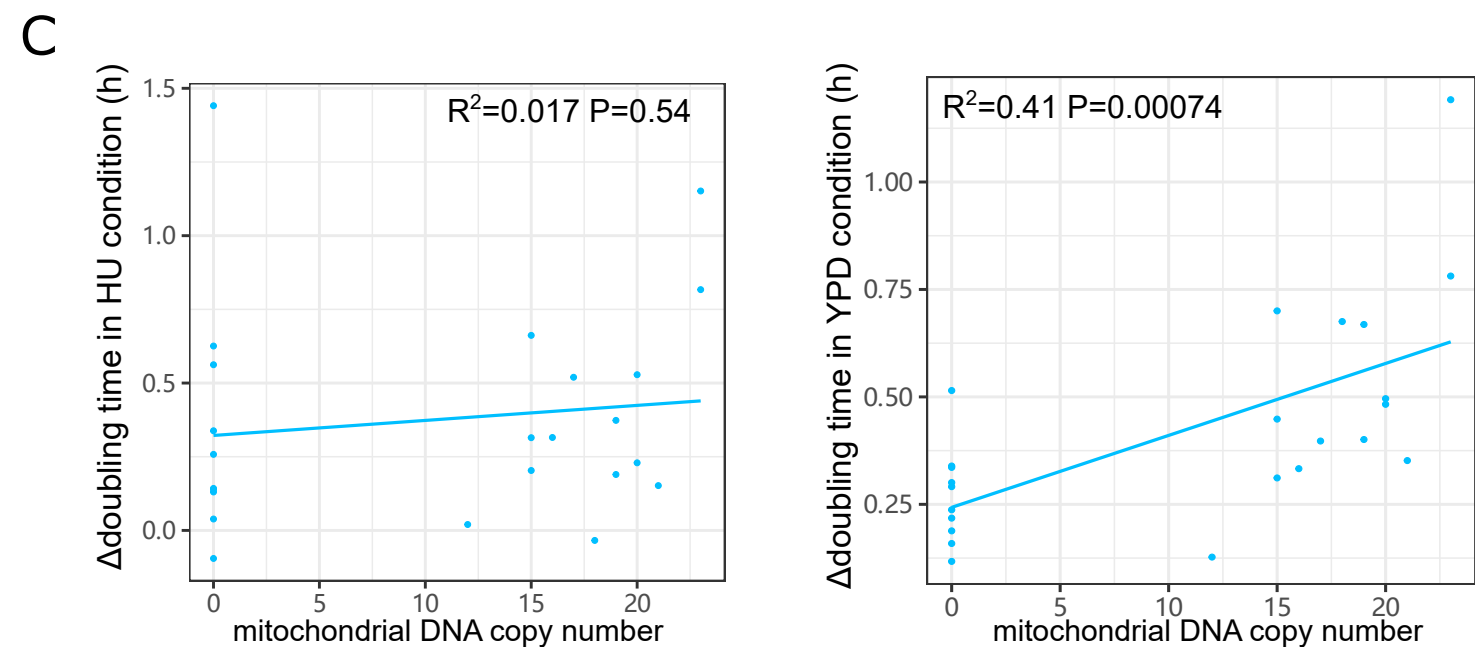

Supplement: S6 Fig — (A) Correlation between whole chromosome CNV rate in HU and doubling time change (last time point—initial time point) measured in HU (left panel) and without HU (right panel). The triangle, square, circle and cross represent MALs with chromosome gain only, loss only, both gain and loss, no whole chromosome CNV. (B) Correlation between substitution rate in HU and doubling time change (last time point—initial time point) measured in HU (left panel) and without HU (right panel). (C) Correlation between mitochondrial DNA copy number in HU and doubling time change (last time point—initial time point) measured in HU (left panel) and without HU (right panel). (PDF) [file pgen.1011012.s014.pdf]

# S7 Fig

A

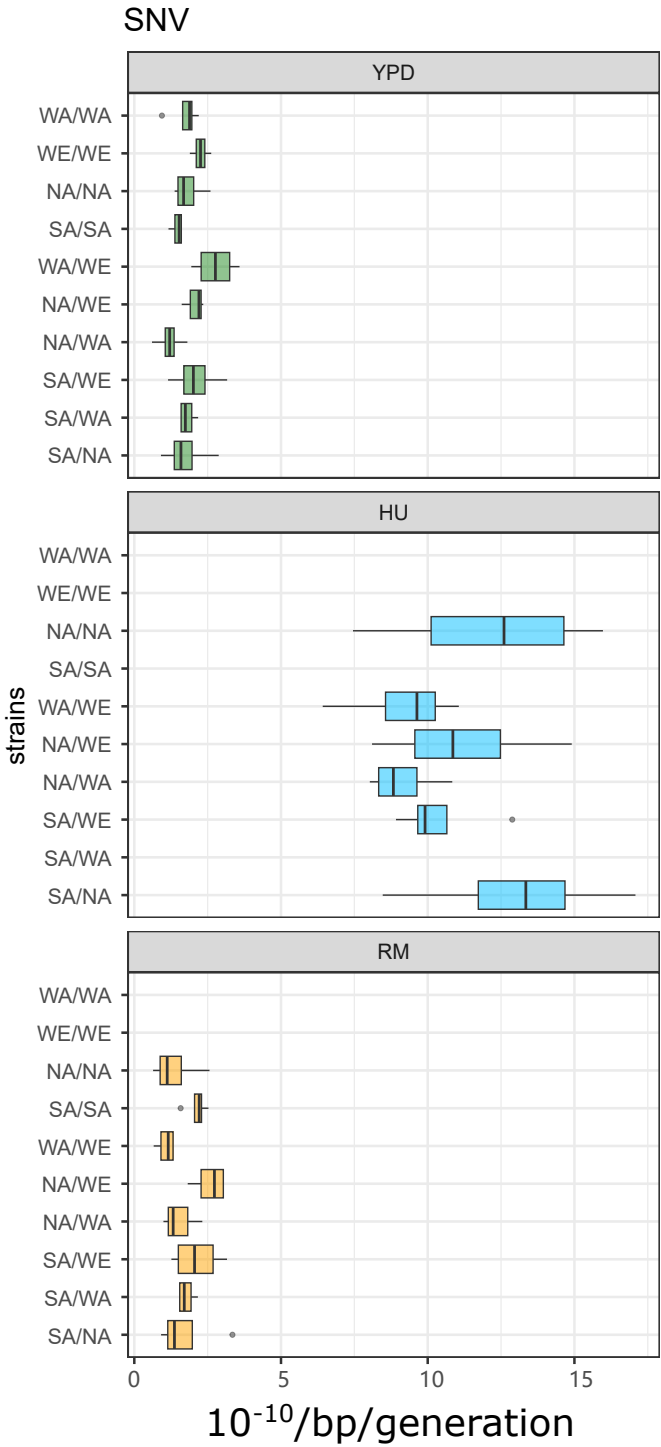

B

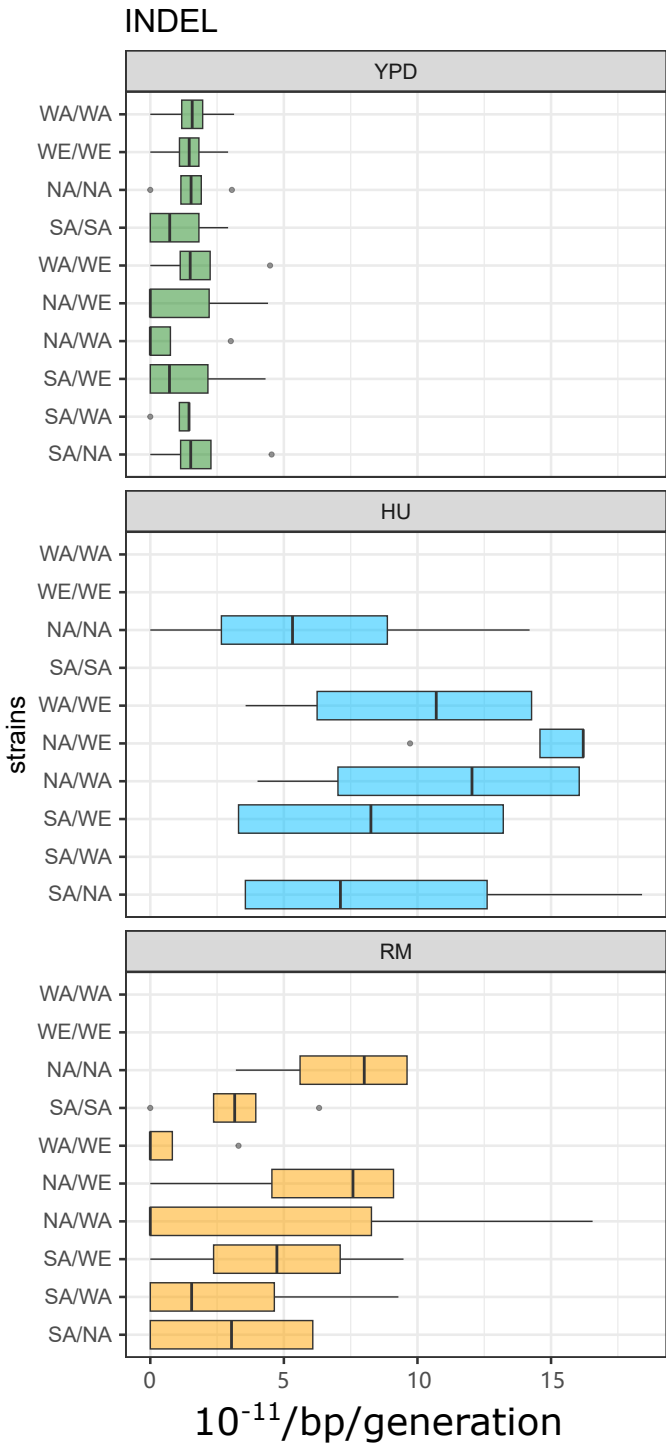

Supplement: S7 Fig — The rate of (A) substitution and (B) INDEL for each MAL partitioned by the genetic background. Each panel from up to bottom shows the mutation rate in YPD, HU and RM condition respectively. (PDF) [file pgen.1011012.s015.pdf]

S8 Fig

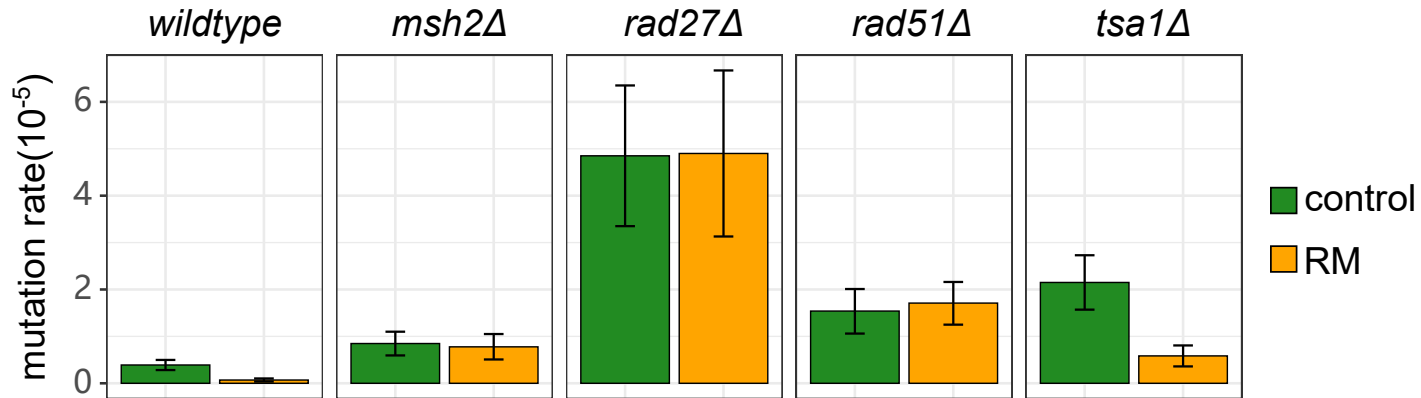

Supplement: S8 Fig — Each strain had 16 replicates. The error bar represents 95% confidence intervals. (PDF) [file pgen.1011012.s016.pdf]

# S9 Fig

A

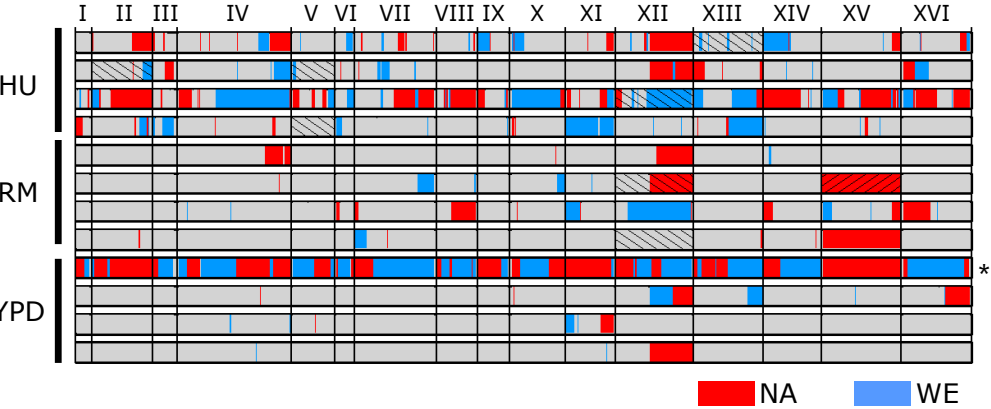

B

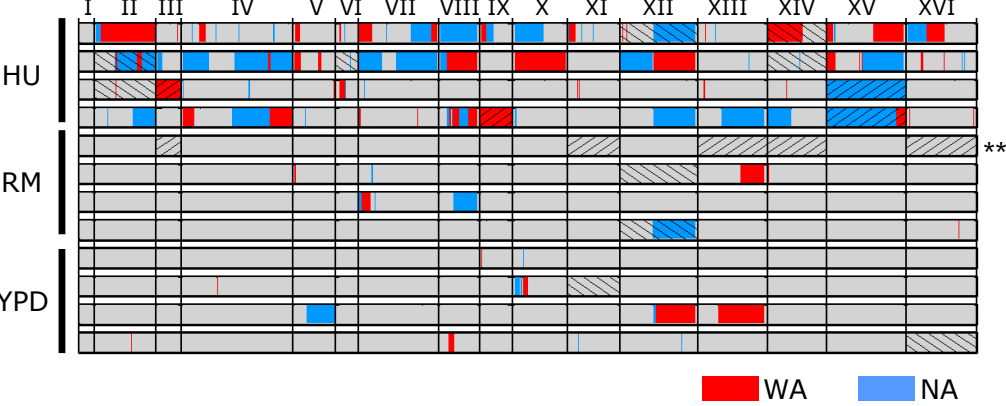

C

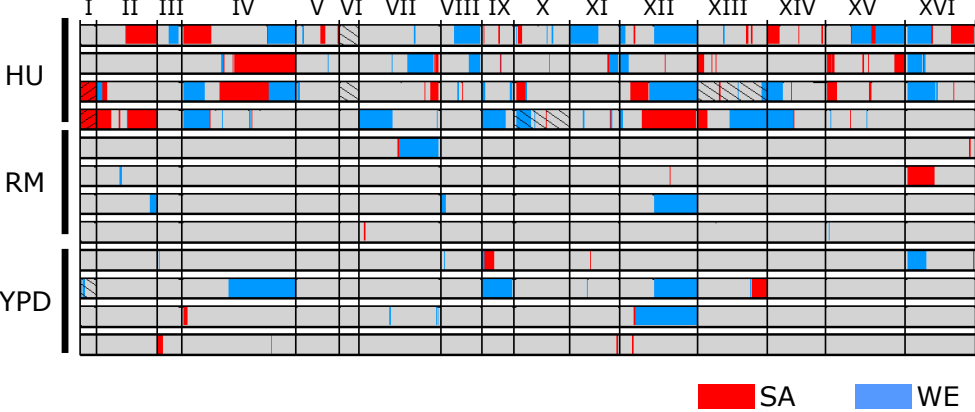

D

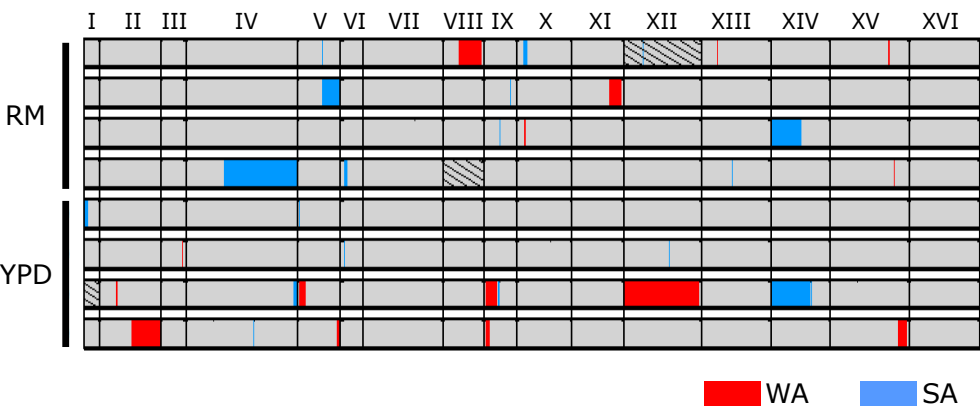

E

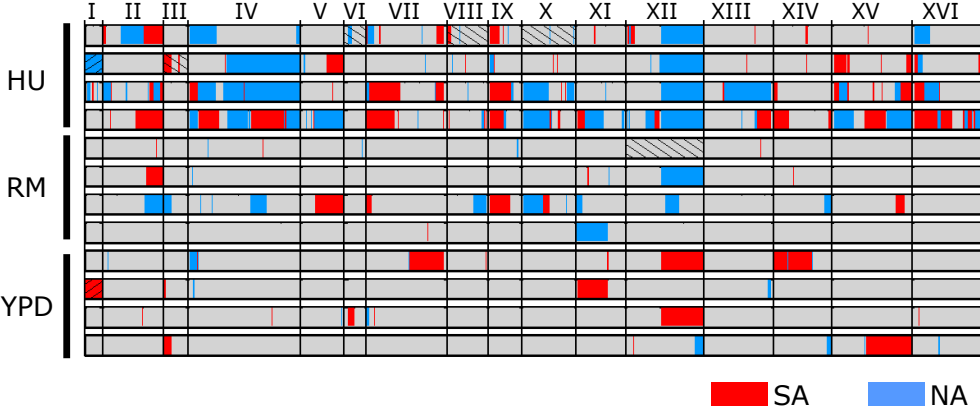

F

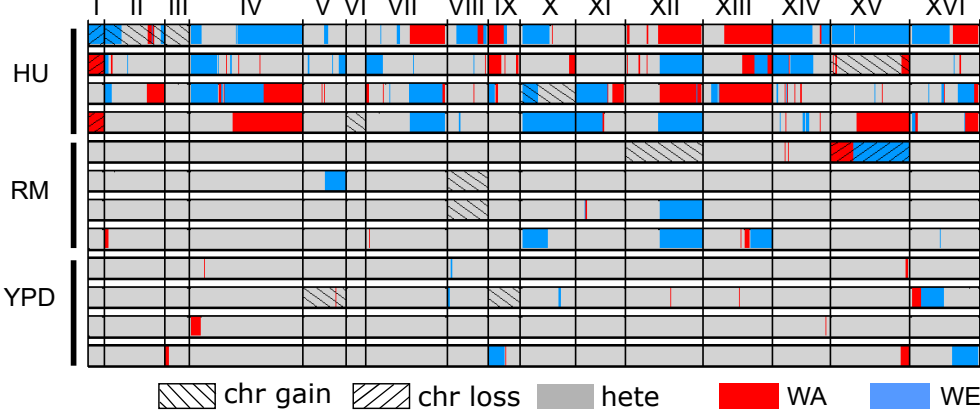

Supplement: S9 Fig — Genome-wide LOH landscape of hybrid mutation accumulation lines (MALs) for (A) NA/WE, (B) NA/WA, (C) SA/WE, (D) SA/WA and (E) SA/NA (F) WA/WE. Each panel shows the LOH landscape of one MAL (from bottom to top represents replicates 1 to 4). The blue and red blocks represent the LOH events towards one of the parents. The grey blocks represent the heterozygous status. The blocks with slash or backslash indicate chromosome loss or gain respectively. (PDF) [file pgen.1011012.s017.pdf]

# S10 Fig

A

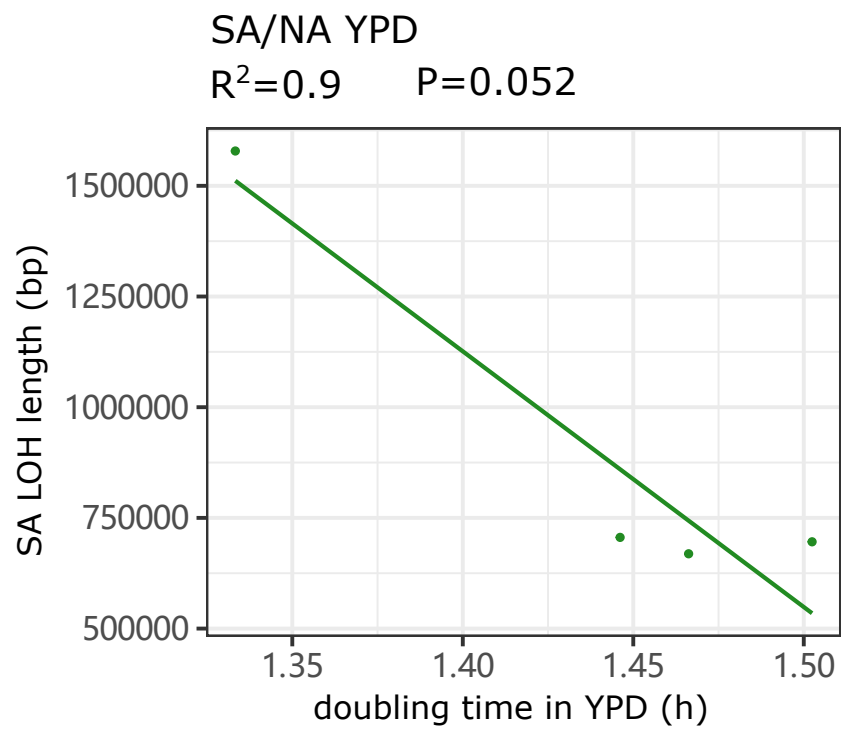

B

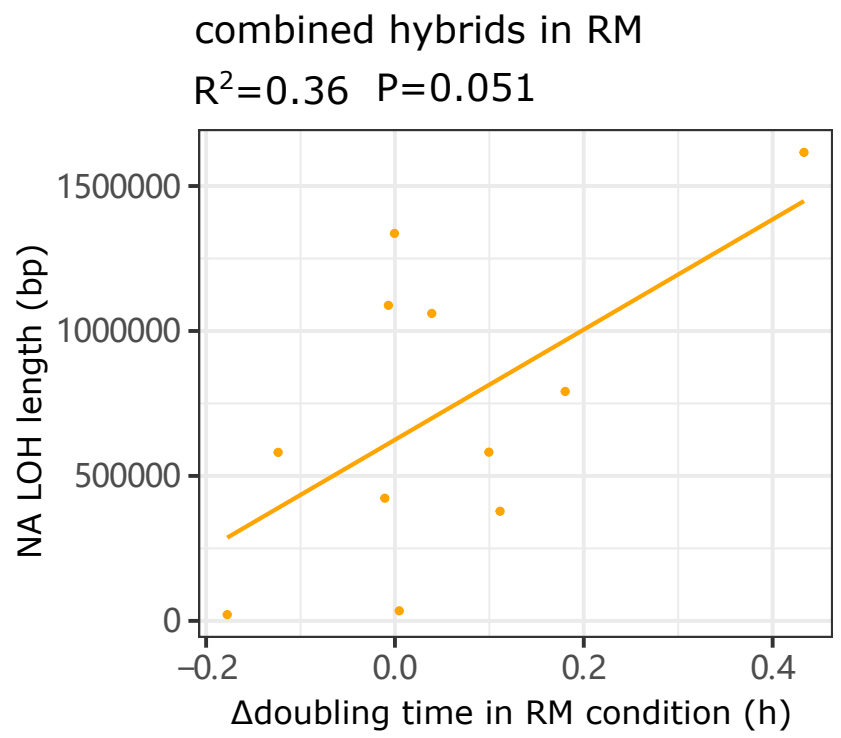

Supplement: S10 Fig — (A) Correlation of the total LOH length of SA allele of SA/NA hybrid (four replicates) and its doubling time in YPD. (B) Correlation of the total LOH length of NA allele of all the hybrids (11 MALs) and their doubling time change in RM. (PDF) [file pgen.1011012.s018.pdf]
