# Supplementary material for: Genome instability footprint under rapamycin and hydroxyurea treatments
Source: PLoS Genet. 2023 Nov 6;19(11):e1011012. doi: 10.1371/journal.pgen.1011012 (PMC10653606; doi:10.1371/journal.pgen.1011012)
Supplement: Attachment — Submitted filename: response_letter.pdf [file pgen.1011012.s019.pdf]

We would like to thank the reviewers for their constructive feedback for our manuscript entitled "Genome instability footprint under rapamycin and hydroxyurea treatments" (PGENETICS-D-23-00878). Here we have changed the manuscript to accommodate the reviewers' viewpoints. A point-by-point response to the reviewers' comments ("replies" [R]) and a detailed description of the corresponding changes made to the manuscript ("actions" [A]) are reported below.

Reviewer #1:

This study is focused on examining the nature of mutational signatures associated with long term environmental/stress conditions. Such an approach would likely be applicable to testing a variety of other environmental conditions. Different S. cerevisiae backgrounds (homozygous and heterozygous) were grown using a mutation accumulation protocol that involved severe (to one cell) bottlenecks every 20 to 23 generations. These studies were performed in rich media (YPD) lacking or containing rapamycin or hydroxyurea. Time-resolved imaging and viability analysis were performed and short and long read DNA sequencing technologies were used to detect base substitutions and indels as well as structural variants in both the nuclear and mitochondrial genomes. The key results were:

1. Long term treatment with rapamycin did not impact the base substitution/indel rate or loss of heterozygosity but led to complete or partial gains in Chr. XII that were associated with reduced rDNA copy number.

2. Treatment with HU resulted in both nuclear and mitochondrial genomes displaying increases in the rate of base substitutions/indels and structural variants. Increases in loss of heterozygosity and aneuploidy were also observed. Analysis of the base substitution/indel spectra showed a correlation with specific error prone repair pathways. These observations are consistent with previous work showing that HU treatment results in replication fork stalling and dNTP starvation.

The genomic and imaging techniques are very well-executed, and the results are convincing. This work follows many studies that have been performed on a large number of DNA repair/replication mutants over a 15-year period. Some of the studies in this field that were particularly comprehensive were published by Lujan et al. (Genome Research 24:1751) and Loeillet et al. (PNAS 117:24947).

[R] [A] We thank the reviewer for summarizing our work and pointing out two key references. We have cited the work of Loeillet et. al. and now added the work of Lujan et. al. in the revised version (line 60): "Yeast MALs have been used to estimate mutation rates and spectra for common lab strains in rich medium[10,11], as well as the effects of controlled variation in ploidy[12–15] and of some specific disruptions in the DNA repair/replication machinery[16–18]."

This study focuses on two sets of exogenous environmental conditions. At least for this reviewer it is not clear if there really is a semantic difference between testing specific environment/stress conditions created exogenously (the addition of a drug) vs. metabolically (cellular mutations that either create damage or prevent repair of damage).

[R] We agree that the mutagenic effect of both exogenous and endogenous factors are important study subjects, i.e., for understanding the root causes of cancer. We also agree that the difference between exogenous and endogenous factors is partially semantic, as the exogenous factors typically act intracellularly with effects that mimic those of endogenous factors. We thank the reviewer for letting us make these clear in the manuscript.

[A] We added text in the Introduction to clarify the exogenous and endogenous factors influencing mutation rates and signature.
Original text (line 40): "Genome instability, referring to the accumulation of mutations, is typified by specific mutational signatures [1]. Such signatures can be shaped by external factors, such as drug treatments, that target different aspects of the cellular machinery."

Revised text: "Genome instability, here referring to the accumulation of both point and large-scale mutations over time, is typified by specific mutational rates and signatures[1]. Genome instability can be shaped by external factors, such as drug treatments that target different aspects of the cellular machinery, and by endogenous factors such as the genetic backgrounds[2]."

Original text (line 66): "Here, we compared the mutation profiles of 96 mutation accumulation lines (MALs), derived from 10 distinct genetic backgrounds, that were evolved for a total of ~181,440 generations in a drug-free (rich medium YPD) condition, stressful hydroxyurea (HU) and rapamycin (RM) conditions. HU and RM represent two distinct mechanisms of action of chemotherapeutic treatments: impairing DNA repair/replication and inhibiting the TOR signaling in proliferating cells, respectively."

Revised text: "Here, we compared the mutation profiles of 96 MALs, derived from 10 distinct genetic backgrounds, that were evolved for a total of ~181,440 generations in a drug-free (rich medium YPD) condition, stressful hydroxyurea (HU) and rapamycin (RM) conditions. The genetic backgrounds (endogenous factors) and the drug conditions (exogenous factors) might both influence mutation rates and signatures. HU and RM represent two distinct mechanisms of action by impairing DNA repair/replication and inhibiting the TOR signaling in proliferating cells, respectively."

There is also some concern about making conclusions for environmental conditions based on a specific drug concentration because other concentrations may result in different phenotypic effects.

[R] We used hydroxyurea (10 mg/ml HU) and rapamycin (0.025 ug/ml RM) at the concentrations that were carefully selected to impede, but not stop, cell proliferation for all the divergent strain genetic backgrounds. Due to the rather narrow dose-response windows of the drugs, and the fact that these windows differ between strains, concentrations cannot substantially deviate from those chosen and still let us perform mutation accumulation

experiments for these genotypes. Moreover, we have previously used these drug concentrations to perform adaptive evolution, i.e., without passing populations through single cell bottlenecks, using the same genetic backgrounds (PMID: 29045840; PMID: 30657986). Using the same drug concentrations here allows us to compare genomic and phenotypic patterns between including and (almost) removing selection as an evolutionary force. For example, in adaptive evolution, we observed frequent *TOR1* and *TOR2* mutations to confer RM resistance. In contrast, in this study (mutation accumulation), we observed frequent chromosome XII amplification as a response to RM treatment but confer no obvious drug resistance. This comparison underscores that selection is indeed minimal in our current MAL experiments, and also underscores the importance of excluding selection through the use of MAL. Nevertheless, following the reviewer's suggestion, we have added caveats to the Discussion section to make it clear to the readers that caution should be employed when extrapolating conclusions across a wider range of drug concentrations.

[A1] We mentioned our previous work using the same drug concentration in the Results (line 84): "The drug concentrations are consistent with our previous adaptive evolution studies of large genetically heterogeneous populations [8,9]."

[A2] We also added text to the Discussion section (line 420): "We used the same drug concentration in the mutation accumulation as in the adaptive evolution that we performed before with the same genotypes [8,9]. The adaptive evolution experiments, which allowed for a substantial impact of selection on evolution, resulted in very different phenotypic dynamics and mutations, underscoring both that our mutational accumulation experiments are not substantially influenced by selection, and that avoiding such an influence is important for drawing conclusions on mutagenic effects. Some caution should be employed when extrapolating conclusions across a wider range of drug concentrations, as drugs often have dose-dependent cellular responses which may impact on mutational rates and signatures[48]."

Regardless, the work is interesting and well-conceived. I have a few comments aimed at improving the manuscript that are listed below.

1. Line 54. How where the single cells to continue the mutation accumulation protocol identified? This seems like an especially important question because many of the conclusions for growth rate were determined using time-resolved imaging techniques. The authors indicated that cells were randomly chosen, but I didn't see any specific details. My reading of the literature indicates that the best approach is to streak a colony to singles, make a mark on the plate in the vicinity of the streak out and then pick for the next bottleneck the colony that forms two days later that is closest to the mark. Indicating a random approach without indicating a specified protocol is a concern.

[R] We thank the reviewer for pointing this out. Our protocol was exactly as described by the reviewer.

[A] We have now better described how the mutation accumulation was conducted in the Methods section (line 496): "For each line, passages were conducted by picking one colony closest to a mark made blindly on the petri dish and then streaking the colony on a fresh plate."

2. Line 159: Were statistics applied to determine if the >2-fold differences were significant?

[R] Yes, we performed the Mann–Whitney U test to compare the aneuploidy rates between YPD and RM conditions, which is significant.

[A] We have added the p-value to the text (line 165): "In RM, the rate of aneuploidies (4.21×10^{-4} events/line/generation) was >2-fold higher than in YPD (p=0.007)". We also added a paragraph of "Statistical Analysis" in the Methods (line 610).

3. Line 209- "…mutation rates and spectra have remained difficult to quantify precisely." Why is this the case?

[R] Mutations can be beneficial, neutral, deleterious or lethal based on their effects on fitness. Most new mutations are thought to be (nearly) neutral, and deleterious mutations greatly outnumber beneficial mutations (PMID: 24166031). However, when mutations occur, those conferring fitness advantages would expand rapidly due to natural selection and hinder the detection of neutral or deleterious mutations. For example, in HU, we only identified *RNR2* and *RNR4* mutations as drivers for HU resistance in the scenario of natural selection (PMID: 29045840; PMID: 30657986). In contrast, with the protocol of mutation accumulation (in this study), the power of genetic drift allows nearly all mutations to accumulate in an effectively neutral fashion (PMID: 27739533), thus, enabling us to investigate the mutation spectra more precisely.

[A] We modified the text to avoid confusion.

Original text (line 219): "HU induced DNA replication stress is widely accepted to lead to rampant genome instability [29,30], but the mutation rates and spectra have remained difficult to quantify precisely."

Revised text: "HU induced DNA replication stress is widely accepted to lead to rampant genome instability[30, 31]. But the mutation rates and spectra have remained difficult to quantify precisely due to the confounding effect of natural selection, which biases studies towards detecting beneficial mutations and against neutral/deleterious ones."

4. Page 229. The authors state that the breakpoints of CNV coincided with rRNA and Ty elements. It's probably worth mentioning the work of Lemoine et al. (Cell 120:587) who found that translocations associated with reduced levels of the replicative alpha DNA polymerase involved homologous recombination between Ty elements.

[R] We thank the reviewer for pointing out the reference, which is now discussed in the relevant results section.

[A] We add the work of Lemoine et. al. as reference (line 240): "The break points of these events coincided with tRNA and Ty elements, consistent with findings from previous genome instability studies of the *S. cerevisiae* deletion collection[33] and the role of Ty in mediating translocations[34, 35]."

Reviewer #2:

The manuscript "Genome instability footprint under rapamycin and hydroxyurea treatments" systematically explored how mutation rate and spectrum and cell growth changes in yeast under rapamycin and hydroxyurea treatments, using different genetic backgrounds. The authors find association of rapamycin treatment with frequent chromosome XII amplifications, but less of an effect on mutation rate. In hydroxyurea treatment, mutation rates are elevated overall with high occurrence of aneuploidy and a mutation bias. In general, this manuscript is clear and well written. The authors have designed their experiments carefully and have done comprehensive analysis with their results. Discovering the mutational effects from different genotypes on these drugs are novel.

It is a lot of undertaken to examine the mutational effects using different genetic backgrounds, and the authors explored both homozygous and heterozygous diploids, with a total of ten genetic backgrounds. It is unclear why the authors choose diploid for this study. Is it because diploids are the most common form in natural populations? Or if the haploid behaviors have been explored by previous studies?

[R] We thank the reviewer for letting us clarify the motivation of using diploid. Our choice was based on the following reasons. First, as the reviewer mentioned, most (~ 87%) of the natural *Saccharomyces cerevisiae* isolates are diploid (PMID: 29643504). Second, diploids are a closer model to humans and would be more relevant for screening therapeutic drugs. Third, using heterozygous diploids allowed us to investigate genome-wide rate and pattern of loss of heterozygosity (LOH), which cannot be observed in haploids (PMID: 33106417). Fourth, in a diploid cell, recessive deleterious mutations, including recessive lethal mutations, are shielded

from selection. Using diploid cells therefore allows us to identify mutations that might be lost in haploid (PMID: 24847077). Fifth, a diploid cell population, all other things being equal, accumulates more mutations than a haploid population, adding statistical power. Taken together, we found the potential advantages of using diploids to be compelling.

[A] We added text in the Results.

Original text (line 79): "To avoid confounding effects from working with a single and potentially atypical genetic background, we selected ten diploid *S. cerevisiae* genetic backgrounds and propagated MALs in hydroxyurea (HU) 10 mg/ml, rapamycin (RM) 0.025 µg/ml and drug-free rich medium (YPD, as control) (Fig. 1a, Table S1)."

Revised text: "To avoid confounding effects from working with a single and potentially atypical genetic background, we selected ten diploid *S. cerevisiae* genetic backgrounds and propagated MALs in hydroxyurea (HU) 10 mg/ml, rapamycin (RM) 0.025 µg/ml and drug-free rich medium (YPD, as control) (Fig. 1a, Table S1). We chose to use diploids because the majority of *S. cerevisiae* natural isolates are found in a diploid state[25], and they are a closer model to higher eukaryotes. As compared to haploids, diploid cells also acquire more and a broader range of mutations (e.g., recessive lethal mutations, chromosome loss and loss of heterozygosity) [11]."

Also, how are the concentration of each drug used in this study determined?

[R] We used hydroxyurea (10 mg/ml HU) and rapamycin (0.025 ug/ml RM) at the concentrations that were carefully selected to impede, but not stop, cell proliferation for all the divergent strain genetic backgrounds. Due to the rather narrow dose-response windows of the drugs, and the fact that these windows differ between strains, concentrations cannot substantially deviate from those chosen and still let us perform mutation accumulation experiments for these genotypes. Moreover, we have previously used these drug concentrations to perform adaptive evolution, i.e., without passing populations through single cell bottlenecks, using the same genetic backgrounds (PMID: 29045840; PMID: 30657986). Using the same

drug concentrations here allows us to compare genomic and phenotypic patterns between including and (almost) removing selection as an evolutionary force. For example, in adaptive evolution, we observed frequent *TOR1* and *TOR2* mutations to confer RM resistance. In contrast, in this study (mutation accumulation), we observed frequent chromosome XII amplification as a response to RM treatment but confer no obvious drug resistance. This comparison underscores that selection is indeed minimal in our current MAL experiments, and also underscores the importance of excluding selection through the use of MAL. Nevertheless, following the reviewer's suggestion, we have added caveats to the Discussion section to make it clear to the readers that caution should be employed when extrapolating conclusions across a wider range of drug concentrations.

[A1] We mentioned our previous work using the same drug concentration in the Results (line 84): "The drug concentrations are consistent with our previous adaptive evolution studies of large genetically heterogeneous populations [8,9]."

[A2] We also added text to the Discussion section (line 420): "We used the same drug concentration in the mutation accumulation as in the adaptive evolution that we performed before with the same genotypes [8,9]. The adaptive evolution experiments, which allowed for a substantial impact of selection on evolution, resulted in very different phenotypic dynamics and mutations, underscoring both that our mutational accumulation experiments are not substantially influenced by selection, and that avoiding such an influence is important for drawing conclusions on mutagenic effects. Some caution should be employed when extrapolating conclusions across a wider range of drug concentrations, as drugs often have dose-dependent cellular responses which may impact on mutational rates and signatures[48]."

What is the purpose to measure cell yield?

[R] Cell yield and cell doubling time represents complementary measures of cell fitness. As an approximation of cell division time, the cell doubling time has a direct connection to fitness and can be directly translated into a selection coefficient. Cell yield, or the number of cells at the end of the experiment, cannot be directly translated into a selection coefficient. Rather, it is

a function of both the length of the lag-phase, which approximates the time to enter the cell division, and the sum of the cell doubling time effect on all cell generations in that growth cycle. Also, because it captures the sum of cell doubling time effects, cell yield gives a larger signal than simply a single cell doubling time, while the measurement precision remains the same, i.e., the signal-to-noise ratio is better.

One issue I had is that although multiple genotypes have been explored, there is no systematic analysis of phenotype for each genotype. It is worthwhile to use quantitative genetics formulas to explore the contribution of genotype, environment, and the interactions on the phenotype of interest, to identify if there are any genotype by environmental interactions.

[R] We found it quite tricky to quantify the genetic contribution of the genotypes, i.e., newly arisen mutations and their potential interactions, because they are confined to a single MAL, and they are present as an aggregate in this MAL. One single exception to this is the amplification of chromosome XII, which occurred in many MALs. For this variant, we have already estimated the fitness effect with and without an environmental interaction term (i.e., with and without drugs). A more detailed view of the genotype-phenotype map would require many re-crossings of individual MALs, either to each other or to their ancestors, and therefore generate a large panel of outcrossed strains. After phenotyping and analysis of the co-segregation of each mutation and each phenotype, the phenotypic contribution of mutations in each background could be estimated. We have previously pursued such an approach for both point mutations (PMID: 29045840, Figure 6) and aneuploidies (PMID: 30657986, Figure 2) in individual genotypes, but to do so across many genotypes constitute a huge experimental effort, which is outside the focus of the current paper.

[A] We added text to clarify this.

Original text (line 265): "The average substitution and INDEL rates in YPD is 1.89×10^{-10} and 1.41×10^{-11} per base per generation respectively (Fig. 4a and 4c, Supplementary Fig. 7, Tables S5-S6), consistent with previous estimates [9–12,21]."

Revised text: "The average substitution and INDEL rates in YPD is 1.89×10^{-10} and 1.41×10^{-11} per base per generation respectively (Fig. 4a and 4c, Supplementary Fig. 7, Tables S5-S6), consistent with previous estimates[10–13,22]. The identified substitutions and INDELs only occurred in single MALs, with each MAL containing multiple mutations, and therefore we cannot directly estimate the phenotypic contribution of each mutation to phenotypes."

For the mutation signature study using COSMIC database (Figure 4), I am not sure how well we can interpret that part of data. Since COSMIC is based on human cancer signatures, it is unknown whether those signatures will be transferrable to interpret yeast mutational processes. It is counter intuitive why SBS18 is enriched in YPD for example (Figure 4e). It seems backwards to me when applying COSMIC mutational signature to infer the biological process, since a lot is known about both RM and HU response pathways in yeast. It will be more straightforward to examine existing studies with mutants in these pathways and see if they lead to expected mutational effects.

[R] We thank the reviewer for letting us clarify the motivation and details to compare the mutational signature with the COSMIC database. Generally, while there certainly are differences between mutational processes in yeast and humans, some important biological processes, like DNA replication and repair underlying many mutation types, are extensively conserved. More specifically, mutational signatures, i.e. Single Base Substitutions, identified in yeast often resemble mutational signatures in human cancers, and can provide important information on the molecular defects in specific tumors (PMID: 32968016). HU and RM cover the two common modes of chemotherapy for treating cancers: inhibition of DNA synthesis (HU) and inhibition of growth (RM). Their primary molecular targets are known and conserved between yeast and humans. Because growth regulation and DNA synthesis are commonly mutated in cancers, it is not unreasonable to expect HU and RM mutational profiles in yeast to resemble mutational signatures in cancers, and this could well hint at these cancers carrying defects RNR and TOR. It is of course also possible that HU and RM have, yet unidentified, off

target effects in yeast, that correspond to mutational processes that are shared with some cancers. When it comes to the SBS18 signature (possibly damage by reactive oxygen species) being present in YPD condition: this signature was not only found in our study but also in the work of PMID: 32968016 and PMID: 29760081, both using yeast as a model. It has been speculated, and we share this speculation, that the signature might be due to the increased ROS production in the population of a late-stationary phase (e.g., two-day) YPD-grown colony (PMID: 15489198).

[A] We added the motivation of the mutational signature analysis.
Original text (line 303): "We compared the mutation patterns observed in our HU evolved genomes to those registered in the Catalogue of Somatic Mutations in Cancer (COSMIC) (Fig. 4e-f)."

Revised text: "To further investigate the mechanisms of HU mutagenesis and establish whether the identified mutational signatures resemble the known mutational characteristics of human cancers, we compared the mutation patterns observed in our HU evolved genomes to those registered in the Catalogue of Somatic Mutations in Cancer (COSMIC) (Fig. 4e-f)."

I have a few minor points:
1. In line 40, genome instability usually refers to large structure changes in the genome, such as chromosome gain or loss and gene copy number changes. Mutational signatures usually capture events at a much smaller scale, although there are associations between the two. The current statement can be a little misleading.

[R] Genome instability is nowadays often used, and used by us in this paper, to refer to an increased frequency of genomic alterations in a broad sense. Therefore, it includes not only large structural changes but also small-scale alterations, such as microsatellite instability and increased frequencies of single base-pair mutations (PMID: 23909437; PMID: 20177397). Mutational signatures, such as those reported in the COSMIC database, encompasses not only

single base substitutions, doublet base substitutions and small insertions and deletions, but also, since 2022, copy number variants (PMID: 35705804). The scale of the latter can be as large as >40 Mb. Thus, as the understanding of genomic evolution evolved, the concepts of both genome instability and mutational signatures have also become broader than they historically had.

[A] We modified the text in the Introduction to clarify this aspect.

Original text (line 40): "Genome instability, referring to the accumulation of mutations, is typified by specific mutational signatures[1]."

Revised text: "Genome instability, here referring to the accumulation of both point and large-scale mutations over time, is typified by specific mutational rates and signatures[1]."

2. The authors referred the mutation accumulation process as "evolution" in the text (e.g. line 97 and 103), which can be misleading. Usually, the term evolution refers to growth in continuous culture or serial dilutions, but not involve serial bottlenecks. "Experimental evolution" usually involves artificial selection.

[R] As systematically reviewed by Barrick and Lenski (PMID: 24166031, also see the figure below), evolution experiments are not restricted to adaptive evolution but can include mutation accumulation for example to study the effect of genetic drift.

[A] In order to avoid confusion, we modified the text.

Original text (line 53): "Asexually reproducing populations of yeast can be propagated in the lab for thousands of generations as Mutation Accumulation Lines (MALs). This experimental evolution protocol minimizes selection by repeatedly forcing cell populations through bottlenecks of random, single cells and mutations are therefore accumulated in a largely unbiased way."

Revised text: “Asexually reproducing populations of yeast can be propagated in the lab by continuous single-cell bottlenecks for thousands of generations as Mutation Accumulation Lines (MALs). This mutation accumulation protocol minimizes selection by repeatedly forcing cell populations through bottlenecks of random, single cells and mutations are therefore accumulated in a largely unbiased way.”

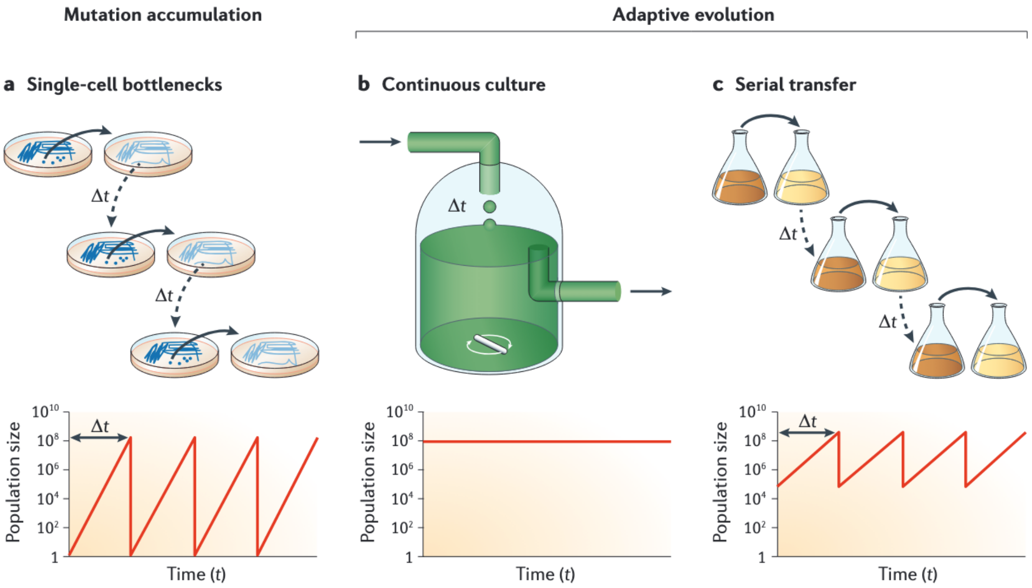

Figure 1 | **Types of evolution experiments.** There are three main ways that populations are propagated in evolution experiments, and they all lead to different types of genetic dynamics. The mechanics of how populations are maintained in each set-up are illustrated for microorganisms (top panels), and representative changes in population sizes over time are also shown for each procedure (bottom panels). Analogous procedures exist for multicellular organisms, although population sizes are generally much smaller. **a** | In mutation accumulation experiments, frequent and deliberate population bottlenecks through one or a few randomly chosen breeding individuals are accomplished by picking colonies of microorganisms that grow from single cells on agar plates. These bottlenecks purge genetic diversity and lead to the fixation of arbitrary mutations without respect to their effects on fitness. **b** | In experiments using continuous culture, populations are maintained in conditions that consist of a constant inflow of nutrients and an outflow of random individuals and waste in a chemostat, which leads to adaptive evolution and genetic diversity in populations that typically maintain a nearly constant size. **c** | In serial transfer experiments, a proportion of the population is periodically transferred to fresh media and allowed to regrow until the limiting nutrient is exhausted. Such batch growth also leads to adaptive evolution because ample genetic diversity is maintained through each transfer. Alternatively, transfers can be made before nutrient depletion, thereby allowing perpetual population growth. A second, cryptic type of population bottleneck occurs during adaptive evolution experiments (parts **b** and **c**) as a consequence of selective sweeps, especially in asexual populations, that drive out competing lineages and thereby reduce genetic diversity.

3. In line 400, it says neither RM or HU imposed selection that confuse conclusion on mutation rates. What is the evidence that supports this statement? Is it based on the viability results at generation 0 (Figure 1b)? Please add the reasoning.

[R] We are sorry for the misunderstanding. We wrote "neither RM or HU imposed detectable selection" because we systematically estimated the phenotypic dynamics across multiple time points during the mutation accumulation (Figure 1c). We found negative or neutral fitness trajectories for populations evolving in HU and RM across random single cell passages, which stands in stark contrast with the strongly positive fitness trajectories of the same lineages when evolving in large cell population sizes under HU and RM selection by serial transfer (adaptive evolution) (PMID: 30657986). Nevertheless, we should avoid the statement of "no selection" because the mutation accumulation approach minimizes the effects of selection - but it cannot eliminate it completely, especially when we apply stresses such as drugs.

[A] We modified the text in the Discussion

Original text (line 416): "Except for an unavoidable selection against lethal mutations, which are removed in the single cell bottleneck steps, neither RM or HU imposed detectable selection that could confuse conclusions on mutation rates on evolving lines."

Revised text: "Except for an unavoidable selection against dominant lethal mutations, which are removed in the single cell bottleneck steps, the negative or neutral fitness trajectories of populations propagated by single-cell bottlenecks (Figure 1c) showed RM and HU imposed limited or no selection on growth, likely with negligible impact on mutation rates."

Reviewer #3:

Li et al. conducted a study focusing on the genomic instability footprint induced by rapamycin and hydroxyurea, two drugs commonly employed in medical treatments. Sequencing only the endpoint of an evolutionary process provides a limited perspective on the spectrum of mutations that have occurred, as natural selection favors beneficial mutations and purges deleterious ones. To overcome this limitation and obtain a comprehensive understanding of the full range of mutations arising during rapamycin and hydroxyurea treatments, the authors used the mutation accumulation assay on a diverse array of S. cerevisiae strains. This approach minimizes the influence of natural selection and enables virtually all non-lethal mutations to accumulate over

time. Through sequencing the final clones, the authors uncovered the genomic instability footprint resulting from rapamycin and hydroxyurea treatment, which manifested as modified frequency and types of aneuploidies, SNPs, INDELs, and loss of heterozygosity events. The meticulous execution of the analysis and its high relevance to the widespread use of rapamycin and hydroxyurea in treatments render this study an important resource for both basic and applied genetics.

Major comments:

Line 173-176: given the potentially significant implications of the aneuploidy of chrXII in response to RM, it would be advisable for the authors to establish causality. To achieve this, they could consider testing the fitness of evolved strains that lack chrXII duplications but have additional rDNA repeats provided as ECRs. Another option is to evaluate the fitness of chr XII aneuploid strains in which a portion of the rDNA array has been deleted.

[R] Following the reviewer's suggestion, we first sorted the RM-evolved MALs by their rDNA copy numbers, as shown in the bar plots below with chromosome XII copy number represented by the bar colors. In general, the MALs with chromosome XII amplification had more rDNA copies. In order to compare their fitness, we plotted the doubling time by dots in the same panel. The same as we said in the manuscript, generally we did not see significant growth advantage or disadvantage among MALs with low or high levels of rDNA. However, when we compared the extreme cases, as the reviewer suggested, we found that the MALs with highest levels of rDNA copies grew faster in rapamycin than the ones with lowest rDNA copies, no matter the MALs with (doubling time 1.90 h vs. 2.15 h) or without (doubling time 1.89 h vs. 2.15 h) chromosome XII duplication (see Table below). Although we cannot draw general conclusions, this indicates that chromosome XII gain did not impose a fitness cost and rDNA copy number was important for the fitness in rapamycin.

[A] We added this analysis in the Results.

Original text (line 184): “However, we found the cell doubling time evolution in RM to be unaffected by chromosome XII copy number while the cell yield gains in RM was marginally better for cells with chromosome XII gain (Fig. 2d).”

Revised text: “However, we found the cell doubling time evolution in RM to be unaffected by chromosome XII copy number while the cell yield gains in RM was marginally better for cells with chromosome XII gain (Fig. 2d). By comparing the fitness of MALs with the highest and lowest rDNA copies, we found that high rDNA copies revealed growth advantage in rapamycin with (doubling time 1.90 h vs. 2.15 h) or without (1.89 h vs. 2.15 h) chromosome XII duplication. Although we cannot make general conclusions from such extreme cases, our data indicates that in rapamycin chromosome XII gain did not result in observable fitness cost and rDNA copy number is important for growth.”

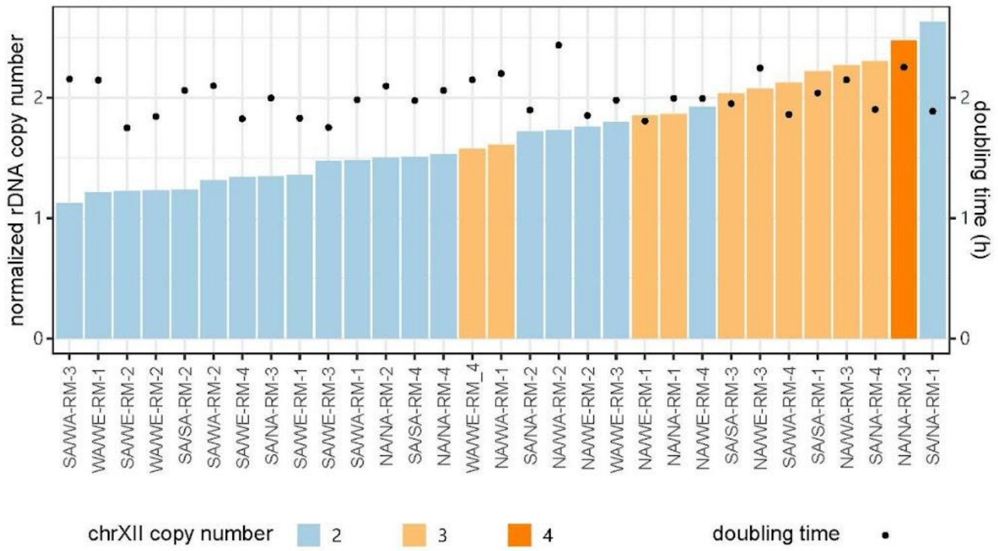

doubling time of MALs (h)	MALs with chrXII duplication	MALs without chrXII duplication
MALs with highest rDNA copies	1.90	1.89
MALs with lowest rDNA copies	2.15	2.15

Minor comments:

Fig4a-c: it could be useful to the reader to specify 'substitutions' and 'indels' when referring to the rates in the plot, on top of writing it in the legend.

[R] [A] We thank the reviewer for pointing out details. We have added annotations of "substitutions" and "INDEL" to the panels of Figure 4a-c.

Reviewer #4:

Li et al perform mutational accumulation of 4 lines from 10 genetic diploid backgrounds (homozygous and heterozygous), each for 2300 (or 1200) generations and in 3 different environments. The goal was to identify a genotype/environment basis for mutation rate and spectra. They identify some differences in the mutational spectra for HU and Rapamycin.

While the work is commendable, it is only somewhat unique in the use of drugs for the mutational accumulation. At the end of the reading, I am not sure what I learned. It is well known that some drugs show distinct mutational spectra, and this can be discovered without the use of long-term MA lines because one can obtain the spectra from hitchhiking mutations on neutral loci (as has been done many times before).

[R] Mutation accumulation experiment is the most unbiased way to investigate mutation rates and spectra, as it minimizes the confounding effects of selection and allows almost all types of mutations to accumulate (PMID: 24166031, PMID: 27739533). Thus, although more laborious, it provides higher resolution, power, and accuracy in the investigation and understanding of the mutational process. This effect is further amplified by expanding beyond a few supposedly neutral loci to a genome-wide perspective, which allow us to investigate not only point mutation and INDELs, but also loss of heterozygosity, structural variation, aneuploidy and other mutations that are far too rare to be capture by single locus studies. The chromosome XII amplifications in RM are the most evident finding in our study that is difficult to be revealed by a single locus study.

The major problem is that there is no dive into mechanism for any of the discoveries they make. The use of the different genotypes is a good control, but they would have learned more about the specific drugs had they used genotypes that had defects in specific mechanisms of HU or Rapamycin stress.

[R] We agree that a further dive into the mutational mechanisms of these two drugs would be interesting, which deserves more dedicated investigations. However, the main question that we want to address with our current manuscript is the general mutational rate and signatures of these two drugs, especially in the contexts of different natural genomic backgrounds. We feel our current angle is equally interesting and does have a general applicable value.

I believe the work is potentially useful for the community, but may be better placed in a more specific journal.

I have a few comments:

1) The sequencing platform, depth, and whether the samples were pooled in a single lane only, is not mentioned in the methods. Without this, it is not possible to assess the substitutions that were detected. The authors use a cutoff of detection of 10x coverage. If the coverage aim was close to 10-20x, then this cutoff can have large false-positives depending on the quality of the sequencing lane. If samples were run on different lanes, then it is simply not comparable.

[R] We thank the reviewer for pointing out missing details of the sequencing approach. We used pair-end (2 x 100 bp) sequencing on the Illumina Novaseq platform at Ginkgo Bioworks (Boston, Massachusetts, United States). The mean sequencing coverage of our samples is 138X. All the samples were sequenced using the same flow cell within the same run. Therefore, the sequencing strategy was fairly applied across samples and should not bring bias for data analysis.

[A] We have added text to the methods section (line 515): "We used pair-end (2 x 100 bp) sequencing on Illumina Novaseq platform at Ginkgo Bioworks (Boston, Massachusetts, United States). The mean sequencing coverage is 138X. All the samples were sequenced using the same flow cell within the same run."

2) It is difficult for me to conclude that the mutational accumulation regime the authors use does not impose selection (at least as argued by the authors). First, RM stress imposes a signature of chr 12 duplication (section starting line 159), which the authors already hypothesize is a selective force due to rDNA contraction. However, rDNA contraction has never been shown to be so deleterious that a contraction of half would be close to lethal and indeed rDNA numbers in the wild vary to numbers much lower than the laboratory strains (~20 copies), and laboratory strains can be made to have such low numbers of copies with decent growth rate. So this would argue against simply selection against lethal mutations (or selective effects of order >10%). Thus, their MA lines have substantial selection against non-lethal mutations.

[R] We partially agree. The mutation accumulation approach minimizes the effects of selection but cannot eliminate it completely, especially when stresses are applied. In addition to lethal mutations, strong deleterious mutations that bring severe growth defects are potentially also selected against. In our case, chromosome XII amplification is an important signature of RM treatment. Several pieces of evidence suggest it is not the target of selection. First, we carefully compared the fitness of clones with and without chromosome XII amplification, but no significant difference was found. Second, our previous work using the same strains (PMID: 30657986) has identified and validated adaptive mutations, such as *TOR1*, *TOR2* and *FPR1*, for RM resistance and no chromosome XII amplification was seen. Third, the proportion of synonymous mutations and genic mutations can be used as a proxy to measure the power of selection (PMID: 31056389). In our case, the proportion of non-synonymous and genic substitutions supported a neutral evolution in RM (observed vs. expected $p>0.33$). Therefore,

although selection cannot be completely excluded, we have carefully utilized the mutation accumulation system to minimize its effects.

[A] We modified the text in the Discussion to avoid a strong statement of no selection and remove the statement of rDNA contraction being lethal.

Original text (line 416): “Except for an unavoidable selection against lethal mutations, which are removed in the single cell bottleneck steps, neither RM or HU imposed detectable selection that could confuse conclusions on mutation rates on evolving lines.”

Revised text: “Except for an unavoidable selection against dominant lethal, or strongly deleterious mutations, which are removed in the single cell bottleneck steps, the negative or neutral fitness trajectories of populations propagated by single-cell bottlenecks (Figure 1c) showed RM and HU imposed limited or no selection on growth, likely with negligible impact on mutation rates.”

Original text (line 440): “Such RM-induced rDNA contractions are possibly near lethal but can be rescued through the clonal amplification of extrachromosomal rDNA circles.”

Revised text: “Such RM-induced rDNA contractions can be rescued through the clonal amplification of extrachromosomal rDNA circles.”

3) Regarding the use of different strains as controls, the WE lines have higher mutation rates. Does this defeat the use of the controls or is this considered a "typical" difference? The authors may want to identify the genetic basis for this mutation rate difference so they can adequately discuss whether this is expected. As it stands, I'm not sure what to make of this observation.

[R] We are sorry for the misunderstanding and thank the reviewer for letting us clarify it. Jiang et. al. reported that natural isolates of *S. cerevisiae* showed a 10-fold range of mutation rates by estimating *CAN1* mutations, indicating the effects of genetic backgrounds on mutation rates

(PMID: 34523420). In our case, we used ten strains with different genetic backgrounds and estimated their mutation rates genome-widely. The highest point mutation rate is 6-fold higher than the lowest in YPD. Therefore, both our results and the published work suggest that the variation of mutation rates in natural strains is quite substantial.

It would indeed be interesting to identify the genetic basis of the mutation rate variation. Doing so, with high accuracy, precision and high confidence in conclusions, would require a very substantial undertaking and would be a paper in itself (PMID: 30504363). We nevertheless took the advantage of our 1002 yeast genome project to find some clues as to what genetic variants that could potentially drive this variation. We searched for WE-specific non-synonymous SNPs that are predicted to be deleterious (score < = 0.05) by SIFT (PMID: 19561590). The associated genes are enriched for the function "sequence-specific DNA binding". These SNPs occur specifically in genes involved in DNA replication and DNA repair, including in *POL12*, *POL2*, *REV3*, *TRM2* and *RFX1*. Although we cannot assess causality solely with the current data, these results indicate that there are at least WE-background-specific defects in genes that are key to a high fidelity of DNA synthesis. Further investigation is needed for validation.

[A] We added text and citation in the Discussion.

Original text (line 428): "However, the spontaneous mutation rate varied somewhat across genetic backgrounds, showing that it is not necessarily perfectly conserved throughout a species and should be generalized with care[23]."

Revised text: "However, the spontaneous mutation rate varied somewhat across genetic backgrounds, showing that it is not necessarily perfectly conserved throughout a species and should be generalized with care[24]. The genetic basis of such mutation variation is not clear but can be investigated by linkage mapping[49]."
